# CD28 co-stimulation induced Lck signaling is important for survival and antigen-specific functionality of re-directed T cells

**DOI:** 10.1186/2051-1426-1-S1-P12

**Published:** 2013-11-07

**Authors:** Pratiksha Gulati, Petra Schuberth, Christoph Renner, Christian Munz, Ulf Petrausch

**Affiliations:** 1 Department of Oncology, University Hospital Zurich, Zurich, Switzerland; 2 Department of Oncology, University Hospital Basel, Basel, Switzerland; 3 Department of Experimental Immunology, University of Zurich, Zurich, Switzerland

## Introduction

Adoptive transfer of re-directed T cells which are engineered to express receptors that target tumor-associated molecules and signal T cell effector functions has emerged as an effective therapeutic approach for cancer. Chimeric antigen receptors (CARs) are recombinant receptors consisting of an antibody derived antigen binding domain coupled to intracellular T cell signaling domains. T cells were genetically engineered with CARs with pre-defined binding and CD28-CD3ζ signaling to initiate T cell activation. CD28 co-stimulation provided by a CD28-CD3ζ CAR is important for T cell activation and persistence mediated by IL-2 secretion. Conversely, IL-2 has also been shown to reduce anti-tumor efficacy by augmenting the survival of Tregs infiltrating tumors. We investigated the impact of the Lck binding moiety in the CD28 CAR endo-domain by comparing CARs with or without Lck signaling.

## Methods

We have generated CAR constructs with single-chain antibody domains recognizing tumor antigens; NY-ESO-1 or Fibroblast activation protein (FAP) linked to CD28-CD3ζ T cell signaling domains. The anti-FAP CAR recognizes surface FAP antigen while the anti-NY-ESO-1 CAR recognizes the NY-ESO-1_157-165_ peptide bound to HLA-A2 on tumor cell. We deleted the Lck binding moiety in the CD28 domain of our CAR constructs. The effect of Lck deletion on antigen specific activation through the CAR was measured by ELISA. Furthermore, phenotypic characterization was performed by flow cytometry.

## Results

As expected, deletion of Lck in the CD28 CAR endo-domain abrogated IL-2 secretion in both CAR constructs. Additionally, we found reduced survival of anti-FAP and anti-NY-ESO-1 re-directed T cells lacking Lck signaling *in vitro*. Furthermore, inhibition of Lck signaling impaired antigen-specific IFN-γ secretion in both CAR constructs. Phenotypically, re-directed T cells lacking Lck signaling displayed increased CCR7 expression compared to cells with intact Lck signaling.

## Conclusion

Thus, we conclude that Lck signaling mediated by CD28 co-stimulation is important to promote the survival by antigen-specific IL-2 secretion. As a surrogate for antigen specific functionality, IFN-γ was analyzed and was found abrogated in the absence of Lck signaling. In future, the efficacy and survival of re-directed T cells with CD28 modification will be studied in a humanized mouse model. This model would provide establishing parts of the human immune system in mice and will be used to test new methods for increasing persistence and anti-tumor efficacy of human CAR+ T cells.

